# Dr. E. Williams

**Published:** 1854-08

**Authors:** 


### DR. E. WILLIAMS.

Our respected foreign correspondent, Dr. E. WILLIAMS, of Cincinnati, has an interesting paper in the *London Medical Gazette*, which we shall transfer to the pages of the next number of our Journal. Dr. WILLIAMS is employing his time abroad most profitably, and makes a favorable impression upon all with whom in his travels he is brought in contact. We have been permitted to make the following extract from a letter to a gentleman in this city, written by Mr. JAMES DIXON, Surgeon to the London Ophthalmic Hospital:

"I am happy of this opportunity of bearing testimony to the unwearied zeal, intelligence, and judgment with which Dr. WILLIAMS is availing himself of the advantages his European tour affords him. Nothing worthy of note seems to escape him, and the shrewd good sense, and thoroughly unprejudiced criticism I have so often noticed in my conversations with him respecting what he had seen in France, are an earnest that his German tour will still farther enrich his stores of professional knowledge.

"We have during the last few years been visited by a great number of our professional brethren from the United States; and it is one of the greatest privileges of our profession, to my mind, to be able, whatever our points of difference may be on political, religious, or other subjects, to hail at once as brothers, on common ground, those who are separated from us, on ordinary occasions, by the width of half the globe. This is a privilege confined to the medical profession, or nearly so."

